# Grief reaction, Depression, anxiety, and coping of relatives after palliative patients’ death in Thailand

**DOI:** 10.1192/j.eurpsy.2023.1856

**Published:** 2023-07-19

**Authors:** T. Tantrarungroj, P. Ocharoen, V. Sachdev

**Affiliations:** 1 Psychiatry; 2 Family medicine, Faculty of Medicine, Ramathibodi Hospital, Mahidol University, Bangkok, Thailand

## Abstract

**Introduction:**

Grief is a normal psychological response in relatives after the loss of their loved ones, which has shown to be associated with psychological reactions like depression, anxiety, and significant stress that many relatives have to cope with. In Thailand, there are limited research studies on grief, especially in palliative settings.

**Objectives:**

To examine grief reaction, depression, anxiety, and coping of relatives after palliative patients’ death.

**Methods:**

A multi-method design was applied. The authors completed the demographic data questionnaire, and the participants finished other measures which included the Hospital Anxiety and Depression Scale (HADS), the Inventory of Complicated Grief (ICG), and the Brief-Coping Orientation to Problems Experienced (Brief-COPE). The qualitative data from the focus group interview was analyzed with thematic analysis.

**Results:**

From the quantitative study, the mean scores of HADS for anxiety and depression subscales were 5.05 and 6.34, respectively, which indicated no anxiety and depressive disorders. The mean score of ICG was 19.51 with highest score on acceptance coping subscale. In contrast, the lowest score was on dealing with the substance subscale. There were significant correlations between anxiety subscale from HADS and ICG (r=0.73), depression subscale from HADS and ICG(r=0.85), and anxiety and depression subscale from the HADS (r=0.79). From the qualitative study, the factors associated with grief reaction could be thoroughly explained according to the perceived character of deceased, perceived character of relatives, relationship characteristics, disease, medical care, and support systems.

**Conclusions:**

The correlations among grief reaction, depression, and anxiety of relatives after palliative patients’ death were high. The grief reaction was associated with many factors, including communication from medical personnel. This finding emphasized the importance of assessing the reactions after loss and associated factors in the relatives after palliative patients’ death. Also, evaluating the ways that the relatives use to cope with their loss, expressing empathy, and supporting the relatives to cope with loss in an adaptive way were recommended.

**Disclosure of Interest:**

None Declared

